# Fishing and temperature effects on the size structure of exploited fish stocks

**DOI:** 10.1038/s41598-018-25403-x

**Published:** 2018-05-08

**Authors:** Chen-Yi Tu, Kuan-Ting Chen, Chih-hao Hsieh

**Affiliations:** 1 Institute of Oceanography, National Taiwan University, Taipei, Taiwan; 2 Institute of Ecology and Evolutionary Biology, Department of Life Science, National Taiwan University, Taipei, Taiwan; 3 Research Center for Environmental Changes, Academia Sinica, Taipei, Taiwan; 4 National Center for Theoretical Sciences, Taipei, Taiwan

## Abstract

Size structure of fish stock plays an important role in maintaining sustainability of the population. Size distribution of an exploited stock is predicted to shift toward small individuals caused by size-selective fishing and/or warming; however, their relative contribution remains relatively unexplored. In addition, existing analyses on size structure have focused on univariate size-based indicators (SBIs), such as mean length, evenness of size classes, or the upper 95-percentile of the length frequency distribution; these approaches may not capture full information of size structure. To bridge the gap, we used the variation partitioning approach to examine how the size structure (composition of size classes) responded to fishing, warming and the interaction. We analyzed 28 exploited stocks in the West US, Alaska and North Sea. Our result shows fishing has the most prominent effect on the size structure of the exploited stocks. In addition, the fish stocks experienced higher variability in fishing is more responsive to the temperature effect in their size structure, suggesting that fishing may elevate the sensitivity of exploited stocks in responding to environmental effects. The variation partitioning approach provides complementary information to univariate SBIs in analyzing size structure.

## Introduction

Size structure plays an important role in maintaining reproductive potential and stability of a fish population. For example, larger individuals tend to produce more and better eggs^1,2^ and have a longer spawning season^3–6^; small and large individuals may spawn at different sites^7–9^. Such bet-hedging strategies provide resilience capacity for populations to sustain under unfavorable conditions^1,10,11^. Hence, investigating the change of size structure may provide insight of how resilient a fish population can be.

Several external forcings may alter the size structure of a fish population. The most well known examples are fishing and temperature. Fishing represents size-selective removal of larger individuals that can truncate the size structure of a fish population^12–15^, which in turn may cause recruitment failure^16^, reduce the reproductive outputs^17^, and increase variability of fish populations^18–20^. It may also lead to evolutionary consequences^21^; for example, the genetic differences found in the populations of Atlantic cod (*Gadus morhua*) from Iceland is due to difference in depth-associated fishing mortality^22^. As such, balanced exploitation^23^ and harvest-slot-limit^24^ have been proposed to prevent fishing-induced size truncation.

Apart from fishing, increasing sea water temperature caused by global warming may also lead to shrinking size structure of marine fish populations^25,26^. Elevated ambient water temperature directly influences fish metabolism at individual level^27^, which increases growth rate and causes earlier maturation at population level^28^. Also, temperature may indirectly influence the recruitment processes through trophic transfer^29^ and thus change the size structure. Based on the match/mismatch hypothesis, the larvae survivorship relates to the match between the timing of larval feeding and the food production^30^. For example, rising temperature since mid-1980s has modified the plankton ecosystem and reduced the survival of young cod in the North Sea^31^. While the fishing and temperature effects have been well documented, their relative contributions on the size structure of fish populations remain poorly understood. This is a critical issue particularly for exploited stocks, because overfishing has been shown to enhance the sensitivity of fish abundance and distribution to climate^32–34^; nevertheless, whether such synergistic effect also occurs in size structure remains relatively unexplored.

Previous studies on quantifying fishing or temperature effects on the size structure of fish populations have been focused on univariate size-based indicators (SBI). Some studies used the upper 95-percentile of the length frequency (L_95_) to test fishing effect^35,36^, while the other used length class diversity to investigate the stability of population through time^37^. Recently, European Commission Marine Strategy Framework Directive required regional (or local) fishery reports to provide the information of basic SBIs (e.g. the mean length) in order to improve the management and maintain the sustainable development^38^. However, it remains unclear whether these univariate SBIs could represent the entire size structure and the status of a population. It has been suggested that no single SBI can represent an effective overall indicator for external forces^39^. Also, SBIs need to be selected carefully based on their implications. For example, L_95_ can only reflect the variety of large fish in fish population. The analysis with the North Sea cod, herring and plaice found L_95_ failed to reveal the effects of external forces on fish populations, as it was rather insensitive in responding to fishing mortality^36^.

To overcome the limitation of existing SBIs, we need an alternative approach to (1) analyze complete information of size structure and (2) examine how external forces affect the size structure. Here, we employed the variation partitioning approach to conduct a size structure-based analysis that examines the variation of size class composition in response to external forcings. Variation partitioning can be best understood as a method for extending multivariate regression. In multivariate regression (y ~ x), y represents a univariate response vector and x represents multiple predictors, x_1_, x_2_, etc. (each is a vector) and possibly their interactions; the contribution of each predictor variable (x_i_) can be evaluated by partial R-square. Whereas in variation partitioning (Y ~ X), Y represents a multivariate matrix and X represents multiple predictors, X_1_, X_2_, etc. (each is a matrix); the contribution of each predictor matrix (X_i_) and their interaction is also evaluated by partial R-square^40^. Variation partitioning is commonly used in community ecology to examine the relationship between species composition (Y matrix) and various sets of explaining variables (e.g. 2 or 3 predictor matrices)^40^. This method has also been extend to analyze temporal and space-time variation of community composition data^41^. Here, we borrow this concept to analyze temporal variation of size composition data in responding to fishing, temperature, and the interaction, with the simplification that fishing and temperature is just a vector. Specifically, for a given fish population, we apply the variation partitioning to quantify how the temporal variation of their size composition responds to fishing, temperature and their interaction (see Methods). The explained fraction of variation (partial R-square) by each factor then allows us comparing their relative contribution in affecting length composition through time.

Next, we perform a cross-stock meta-analysis linking the relative contribution of fishing or temperature (the output of variation partitioning as explained fraction of variation) to the life history traits of fishes, as well as long-term mean and variability of fishing or temperature (see Methods). This meta-analysis aims to examine which factor can explain the relative contribution of fishing, temperature and their interaction across stocks. This meta-analysis is motivated by the fact that life history traits are associated with the size structure of population^42^. We hypothesize that the large, slow growth, and late-matured species is more likely to be impacted by fishing in their size structure because size-selective removal (i.e. size truncation) may be more severe in these species^43^ and their recovery will take longer time^42,44^. We also hypothesize that small species is more vulnerable to temperature effects, because smaller species are more sensitive to temperature changes due to the constrains from metabolic allometries^45^.

Furthermore, we expect that fishing and temperature might exhibit interactive effects via multiple ways^46,47^. For example, the long-term fishing effects, such as long-term mean and variability of mortality ratio (fishing mortality divided by natural mortality, *F/M*) may affect the relative contribution of temperature in explaining temporal variation of size structure. Here, we standardize fishing mortality by natural mortality in order to have a fair cross-stock comparison. Motivated by previous studies showing that fishing elevated sensitivity of exploited stocks to environmental changes^18,19,43^, we hypothesize that the fish stocks experienced higher fishing pressure is more responsive to temperature effect in their size structure. We also hypothesize that habitat conditions, including mean and variability of temperature, affect the relative contribution of fishing effect. For instance, Wang *et al*.^48^ found that temperature affects the cod’s life history trait, making the cod population more vulnerable to fishing.

Our objectives are, first, to apply variation partitioning to quantify how the variation of size structure responded to fishing, temperature and the interactive effects for 28 exploited stocks (Table 1) living in a wide range of habitats, including the west coast of US, Alaska, and North Sea (Figure S1). Secondly, we linked the fraction of explained variation by fishing (or temperature) to life history traits (including von Bertalanffy growth rate (K), length infinity (L_inf_), age at maturation (A_50_), length at maturation (L_50_)), as well as long-term mean and variability of fishing and habitat temperature conditions. Finally, to demonstrate the efficacy of our approach, we compared the performance of variation partitioning approach with the traditional univariate SBIs analyses. This comparison is straightforward, as both the univariate SBIs analysis and variation partitioning are computed using similar linear modeling of variance/covariance, with the difference only in the response variable- the response variable is a vector (y) in the univariate SBIs analysis whereas the response variable is a matrix (Y) in the variation partitioning; two methods have the same explaining variables (i.e. fishing and temperature).

**Table 1:** Data regions and periods for the 28 commercial stocks. The abbreviation in the bracket indicates the location of stock: AI, Aleutian Islands; EBS, East Bering Sea; GOA, Gulf of Alaska.

Area	Species	Common Name	Data period	Data length (df)
West US	*Atheresthes stomias*	Arrowtooth flounder	1986–2006	20
West US	*Sebastes goodie*	Chilipepper rockfish	1978–2006	28
West US	*Sebastes crameri*	Dark blotched rockfish	1977–2008	31
West US	*Microstomus pacificus*	Dover Sole	1966–2004	38
West US	*Parophrys vetulus*	English Sole	1965–2008	43
West US	*Ophiodon elongatus*	Lingcod	1965–2008	43
West US	*Sebastolobus altivelis*	Longspine thornyhead	1981–2003	22
West US	*Eopsetta jordani*	Petrale sole	1966–2008	42
West US	*Sardinops sagax*	Sardine	1981–2008	27
West US	*Sebastes diploproa*	Splitnose rockfish	1978–2008	30
West US	*Sebastes ruberrimus*	Yelloweye rockfish	1978–2007	29
Alaska (AI)	*Gadus chalcogramma*	Walleye pollock	1983–2006	9
Alaska (GOA)	*Gadus chalcogramma*	Walleye pollock	1984–2009	24
Alaska (EBS)	*Hippoglossoides elassodon*	Flathead sole	1982–2010	28
Alaska (GOA)	*Hippoglossoides elassodon*	Flathead sole	1984–2009	11
Alaska (EBS)	*Gadus macrocephalus*	Pacific cod	1982–2009	27
Alaska (GOA)	*Gadus macrocephalus*	Pacific cod	1984–2009	11
Alaska (GOA)	*Glyptocephalus zachirus*	Rex sole	1978–2007	11
North Sea	*Gadus morhua*	Cod	1977–2014	37
North Sea	*Melanogrammus aeglefinus*	Haddock	1977–2014	37
North Sea	*Clupea harengus*	Herring	1977–2014	38
North Sea	*Scomber scombrus*	Mackerel	1980–2014	34
North Sea	*Trisopterus esmarkii*	Norway pout	1984–2014	30
North Sea	*Pleuronectes platessa*	Plaice	1977–2014	37
North Sea	*Pollachius virens*	Saithe	1977–2014	37
North Sea	*Solea solea*	Sole	1977–2014	37
North Sea	*Sprattus sprattus*	Sprat	1977–2014	37
North Sea	*Merlangius merlangius*	Whiting	1990–2014	24

## Results and Discussion

**Figure 1:**
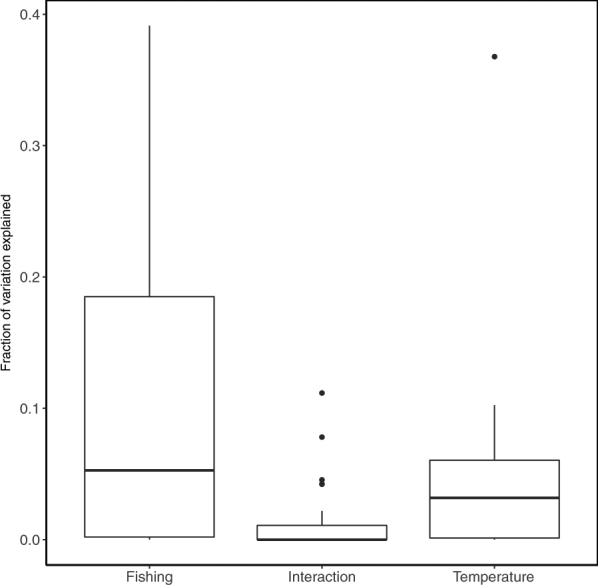
Boxplot showing the variation of size structure explained by the fishing, temperature, and interactive effect. Results of ANOVA indicate that the fraction of variation explained by fishing is significantly higher than the temperature (*P* = 0.019) and interactive effect (*P* = 0.00023), but the fraction of variation explained by temperature is not significantly different from interaction (*P* = 0.344).

Our results of variation partitioning showed that the variance of size structure could be appreciably explained by fishing (on average of 10.9%), which is significantly higher than that of temperature and interaction (*P* = 0.019 and *P* = 0.00023 in ANOVA with paired t-test) (Fig. 1). Specifically, 12 out of 28 stocks were significantly affected by fishing while 7 stocks were significantly associated with temperature (Table 2); whereas, the interactive effect is small in most of stocks (Table 2).

**Table 2:** Results of variation partitioning showing the relative contribution of fishing, temperature, and their interactive effect to the total variation (in term of adjusted R^2^ value) for each of the 28 stocks.

Area	Species	Fishing	Interaction	Temp.	Total adjR^2^
West US	*Atheresthes stomias*	0.211**	0.000	0.070*	0.262^‡^
West US	*Sebastes goodie*	0.176**	0.000	0.000	0.173
West US	*Sebastes crameri*	0.012	0.000	0.030	0.029^‡^
West US	*Microstomus pacificus*	0.038	0.001	0.094*	0.134
West US	*Parophrys vetulus*	0.000	0.005	0.021	0.009^§^
West US	*Ophiodon elongatus*	0.214**	0.000	0.058**	0.253^‡^
West US	*Sebastolobus altivelis*	0.391**	0.000	0.006	0.392^‡^
West US	*Eopsetta jordani*	0.093**	0.000	0.005	0.093
West US	*Sardinops sagax*	0.033	0.000	0.035	0.057^§^
West US	*Sebastes diploproa*	0.122*	0.000	0.015	0.110
West US	*Sebastes ruberrimus*	0.312**	0.045	0.040	0.398^§^
Alaska (AI)	*Gadus chalcogramma*	0.000	0.000	0.368	0.266
Alaska (GOA)	*Gadus chalcogramma*	0.000	0.042	0.000	0.000
Alaska (EBS)	*Hippoglossoides elassodon*	0.000	0.000	0.064	0.025^‡^
Alaska (GOA)	*Hippoglossoides elassodon*	0.134*	0.000	0.000	0.109^§^
Alaska (EBS)	*Gadus macrocephalus*	0.071*	0.000	0.034	0.104^§^
Alaska (GOA)	*Gadus macrocephalus*	0.003	0.022	0.000	0.000
Alaska (GOA)	*Glyptocephalus zachirus*	0.312	0.000	0.059	0.226
North Sea	*Gadus morhua*	0.067*	0.078	0.094**	0.239^§^
North Sea	*Melanogrammus aeglefinus*	0.139**	0.000	0.011	0.116^‡^
North Sea	*Clupea harengus*	0.003	0.009	0.001	0.013^§^
North Sea	*Scomber scombrus*	0.000	0.011	0.000	0.000
North Sea	*Trisopterus esmarkii*	0.000	0.000	0.001	0.000
North Sea	*Pleuronectes platessa*	0.292**	0.112	0.037*	0.440
North Sea	*Pollachius virens*	0.027	0.042	0.037	0.106
North Sea	*Solea solea*	0.000	0.012	0.072*	0.078^‡^
North Sea	*Sprattus sprattus*	0.025	0.000	0.102**	0.108
North Sea	*Merlangius merlangius*	0.372	0.000	0.001	0.351^§^

*Indicates *P* < 0.05; **Indicates *P* < 0.01; ^§^Indicates maximum adjusted R^2^ at 1-year lag; ^‡^Indicates maximum adjusted R^2^ at 3-year lag.

**Figure 2:**
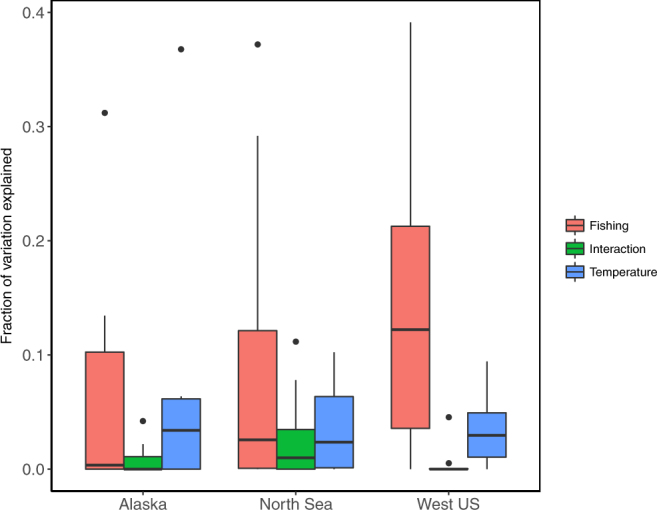
Boxplot showing the variation of size structure explained by the fishing, temperature, and interactive effect grouped by areas. The fraction of variation explained by fishing is significantly different from both temperature (*P* = 0.005) and interaction (*P* = 0.00046) in West US.

**Figure 3:**
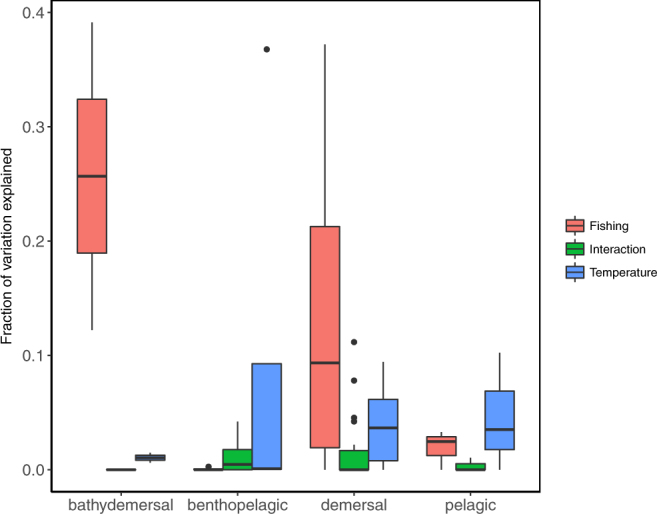
Boxplot showing the variation of size structure explained by the fishing, temperature, and interactive effect grouped by the habitat of species. Particularly, the fraction of variation explained by fishing is significantly different from both temperature (*P* = 0.0014) and interaction (*P* = 7.5 × 10^−5^) for demersal species.

We also found difference in fishing and temperature effect among regions (Fig. 2) and habitat types (Fig. 3). The fraction of variation explained by fishing is significantly different from temperature (*P* = 0.005) and interaction (*P* = 0.00046) in the west US. Both fishing and temperature effects are not significantly different from interaction in Alaska (*P* = 0.74 and *P* = 0.73) and North Sea (*P* = 0.27 and *P* = 1) (Fig. 2). Among habitat types, fishing effect on size structure was significantly different from both temperature and interaction for demersal species (*P* = 0.0014 and *P* = 7.5 × 10^−5^, ANOVA with paired t-test) (Fig. 3).

We employed the variation partition approach to analyze the time-series data of size structure of exploited stocks and found that fishing effect on size structure prevails (Table 2). Such commensurate result goes with the study that analyzed the stock biomass of 28 stocks in the Northeast Atlantics^32^; that is, in a large-scale, fishing is the most critical factor. Our analyses echo the increasing concern over effects of fishing on size structure of exploited stocks^13,26,47,49^.

**Figure 4:**
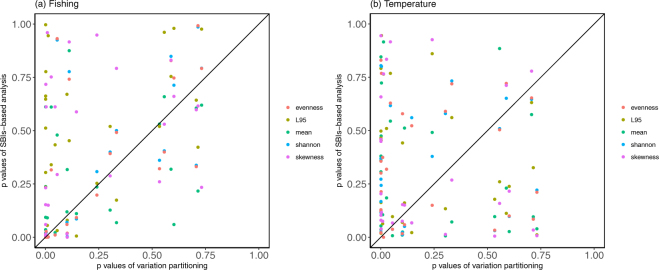
Comparison of *P* values of (**a**) fishing and (**b**) temperature effect estimated from the variation partitioning approach versus univariate SBIs. The diagonal line represents the 1:1 line. Results of binomial test indicate that variation partitioning is more efficient in rejecting the null hypothesis (p-value is smaller) than the univariate SBIs in detecting temperature effects (Probability of success = 0.63, *P* = 0.003) and marginally in fishing effects (Probability of success = 0.56, *P* = 0.10).

We note however, many of these studies applied univariate size-based indicators (SBIs) as proxies for the change of fish size structure. Our approach incorporates the full size structure information in the analysis without assuming distribution of size data. Comparison between variation partitioning and univariate SBIs suggests that variation partitioning is more efficient in rejecting the null hypothesis (p-value is smaller) than the univariate SBIs (Fig. 4), particularly in detecting temperature effects (Probability of success = 0.63, *P* = 0.003) although only marginally in fishing (Probability of success = 0.56, *P* = 0.10). In other words, variation partitioning can be a useful complementary method to investigate the external forcings on the size structure of fish populations, in addition to univariate SBIs.

**Figure 5:**
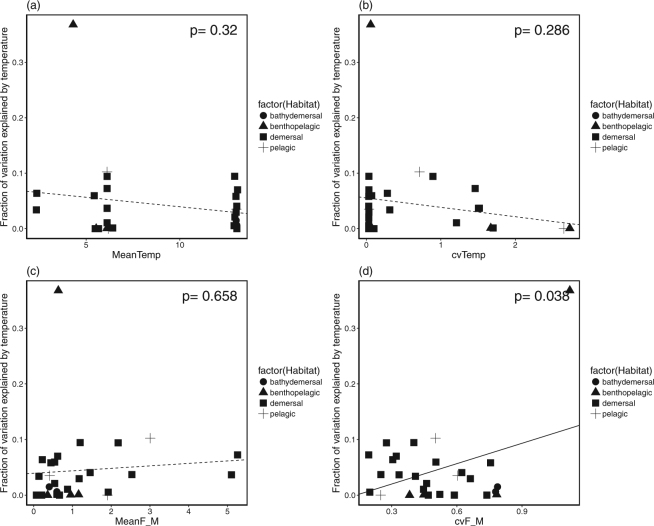
Explained variation by temperature in relation to the mean and CV of mortality ratio or temperature. The line is the best-fitted regression line based on the linear mixed effect model with each fishing/temperature index as fixed effect and habitat as random effect. The solid line indicates the significant result (**d**).

Our multi-stock meta-analytical framework also allows us to investigate how life history traits, and long-term mean and variability of fishing and temperature influence on the explained variation of size structure responding to fishing or temperature. The stocks experienced higher fishing variability (CV of mortality ratio) is more responsive to the temperature effect in their size structure (Table 3, Fig. 5d, *P* = 0.038). This supports our hypothesis that fishing elevates the sensitivity of exploited stocks in responding to environmental changes.

**Table 3:** Results of univariate linear regression analysis on % variation explained by fishing or temperature versus each life history trait, mean mortality ratio (meanF_M), CV of mortality ratio (cvF_M)), mean temperature (meanTemp), and CV of temperature (cvTemp).

		A_50_	L_50_	L_inf_	K	meanF_M	cvF_M	meanTemp	cvTemp
Fishing	coeff.	0.014	−0.001	0.000	−0.089	−0.004	0.075	0.009	−0.022
	*P* value	0.125	0.622	0.596	0.509	0.840	0.511	0.163	0.459
Temperature	coeff.	0.000	0.001	0.000	0.040	0.004	0.122	−0.003	−0.017
	*P* value	0.975	0.523	0.927	0.599	0.673	0.048	0.343	0.308

Surprisingly, we found none of the life history traits is able to explain the fishing and temperature effect (Figures S4, S6). Also, the total adjusted R^2^ (Table 2) suggests that fishing plus temperature explained at most 44% of variation among 28 stocks. There may be other factors associated with body size, such as oxygen limitation of thermal tolerance^50^, affect the response to the fishing/temperature effect on the size structure.

Through the application of variation partitioning, we had expected the efficacy to identify interactive effect of fishing and temperature on the size structure. However unexpectedly, our results indicate that the interaction effect is very weak (Fig. 1). This finding may superficially be interpreted as evidence for lack-of interaction of fishing and temperature on size structure because fishing effects have dominated. However, we caution the interpretation of this finding, as variation partitioning is a linear variance decomposition method, which cannot account for nonlinear interactions.

While we demonstrate the efficacy of our size-structure based, meta-analytical framework to examine fishing and temperature effects on size composition of exploited stocks, we shall point out some caveats in our study. First, fishery-dependent data may lose some information due to the discard of small-size individuals. Second, there were fewer pelagic species than demersal and benthic species in our dataset, which might cause biased interpretation. Third, we assume an instantaneous response or a fixed lag in order to relate changes in size structure to changes in temperature, and we cannot provide detailed information concerning size class-specific responses. This may be an important concern, because, for example, a warm year may lead to a strong year class and therefore affect the size structure of this year^25,51,52^, whereas the effect of temperature on the asymptotic body size may occur for several years.

## Final Remark

We introduced the size-structure based approach relying on variation partitioning to quantify fishing and temperature effects on size composition of exploited fish stocks, instead of focusing on univariate size-based indicators. Through our multi-stock meta-analytical framework, we found that fishing explained most of the variation (Fig. 1), but difference existed between different regions (Fig. 2) and habitats (Fig. 3). We acknowledge that our analytical method still assumes linear responses of size structure to external forcings (as all univariate SBIs analyses do), because the nonlinear response forms remain unknown. Nevertheless, our analytical framework is a step toward better quantification of fishing and temperature effects on the size structure of exploited stocks in the context of life history theory. The information gained here may be useful for ecosystem-based approaches to fisheries.

## Material and Methods

### Size structure data of commercial stocks

We collected length frequency data from 28 exploited stocks, which contain temporal coverage over 20 years and annual fishing mortalities (or exploitation rates) estimated by stock assessment are available (Tables 1, S1). These stocks came from 3 regions in the northern hemisphere (Figure S1): (1) the west coast of US (West US), which is part of the Northeast Pacific; (2) the Alaska region, which separates into 3 fishing areas- the Aleutian Islands (AI), Gulf of Alaska (GOA), and Bering Sea; and (3) the North Sea. Collectively, these 28 stocks in 3 regions were well studied, spanning distinct distribution of size structure, with a wide range of life-history traits and habitats, and therefore are representative of a compilation of global-scale fish stocks (Table S2).

We primarily use length frequency from the fisheries-independent surveys for each stock. These are the length frequency per size range of the given year as arranged in the stock assessment reports. For the Alaska region, the survey data include the bottom trawl surveys in the Aleutian Island (AI), East Bering Sea shelf (EBS) and Gulf of Alaska (GOA). For the North Sea, we compiled the length frequency data of the ICES International Bottom Trawl Survey in the North Sea (NS-IBTS). We used the 1^st^ quarter (Q1) in the NS-IBTS for consistency because there was only annual Q1 survey prior to 1991. For the west coast of US, we used fisheries-dependent length compositions instead of bottom trawl survey because the data from fishery-independent surveys during 1980–1990 were limited.

### Fishing and natural mortality

To quantify the fishing effect, we used time series of annual fishing mortality from the stock assessment reports (Table S1). We focus on the single fishery whenever possible to minimize the uncertainty in fishing selectivity due to changing gears. We noted that analyses in the west coast of US use exploitation rate instead of fishing mortality in stock assessment. To make a fair comparison for meta-analysis, we transformed the exploitation rate into fishing mortality through the relationship between mortality and survival rate in fisheries^53^. Here, we first assume that these fisheries are type II fishery, in which the fishing and nature mortality operate concurrently. The exploitation rate (*μ*), fishing mortality (*F*), natural mortality (*M*), instantaneous total mortality rate (*Z*), and actual total mortality (*A*) have the following relationships:

1 $$\mu =F\cdot A/Z$$

2 $$Z=F+M$$

3 $$A=1-{e}^{(F+M)}$$

The equation (1) can also be written as:

$$\mu =F\cdot (1-{e}^{(F+M)})/(F+M)$$

With the known exploitation rate and natural mortality, this equation can be solved numerically and yields the fishing mortality. The natural mortality here is mostly compiled from the value of the preferred model in the stock assessment reports (Tables S1, S2).

### Temperature

In analysis, we primarily used empirical measurements of temperature along with the trawl surveys (see Figure S2). The North Sea (53–59°N, 3°W–10°E) near-bottom temperature is the station observations of hydrochemical measurements from the ICES Oceanographic database (http://ocean.ices.dk/HydChem/HydChem.aspx) at Q1 (January-February), with coverage of almost the entire North Sea. For the Alaska region (Aleutian Islands, East Bering Sea and Gulf of Alaska) the bottom trawl surveys provide bottom temperature measurements (https://www.afsc.noaa.gov/RACE/groundfish/survey_data/data.htm). We took average for all the stations that the species occurred. Although many demersal species examined here have a pelagic phase at larval-juvenile stages and some species (e.g. Atlantic cod) forage on the whole water column, the preliminary analysis on surface (see Figure S3) and bottom temperature suggests that they are highly correlated (AI: 0.92, EBS: 0.72, GOA: 0.67, North Sea: 0.76). This suggests even the water is stratified, the interannual variability is very similar in the temperate-subarctic ocean. Therefore, we use only the bottom temperature collected in trawl surveys for these 4 areas in our analysis. For the west coast of US, we used the sea surface temperature from the NCEP/NCAR Re-analysis monthly mean (http://www.esrl.noaa.gov/psd/data/timeseries/) and then took aerial average (31.4–48.6°N and 54.4–56.3°W) to represent the entire region.

### Variation partitioning to quantify fishing and temperature effect on size structure

To determine how much of the temporal variation in size structure is explained by the fishing and temperature effect, variation partitioning (*aka*. redundancy analysis) was used to decompose the total variation of length composition of each stock through years. The response variable here is the matrix of size composition data through time. The explanatory variables were time series of annual fishing mortality and temperature. The unbiased estimation of adjusted R^2^ (accounting for sample size effect) provides a test with correct Type I error rate and good power for redundancy analysis^40^. We reported the adjusted R^2^ for the pure effect of fishing, temperature and their interaction. Next, we perform permutation test (1000 times) to evaluate the significance of each fraction. Although the significance test cannot be done for the interactive component^40^, we still report the interactive component for the sake of comparison.

To incorporate the lagged effect of temperature, we additionally used 1 year- and 3 year-lag temperature as explanatory variables in variation partitioning. For the stocks in the Alaska region, sampling interval is 2- or 3-year, and thus we only considered 2 or 3-year lag. We did not investigate lagged effect of fishing; we assumed that fishing instantaneously affects the adult population while temperature may affect the size structure through influencing the future recruitment at early life stages (e.g. egg and early larvae). Because variation partitioning does not estimate log-likelihood in the procedure, the best model is selected according to the largest effective size (highest total adjusted R^2^ (see Table 2)). The fraction of variation explained by fishing and temperature from the best model was used in further analyses.

### Variables affecting the relative contribution of fishing and temperature effect

To investigate what determines the relative contribution of fishing and temperature across stocks, we considered the following variables (see Table S2): (1) life history traits: von Bertalanffy growth rate (K), length-at-infinity (L_inf_), age at maturation (A_50_), and length at maturation (L_50_); (2) indices of fishing effect: long-term mean and variability of mortality ratio (i.e. fishing mortality/natural mortality, *F/M*); and (3) indices of temperature effect: long-term mean and variability of temperature. We used the mortality ratio (*F/M*) to reflect the fishing strength among different stocks. Because fisheries management usually sets optimal fishing mortality (*F*_*opt*_) proportional to natural mortality (*M*)^54^, here we defined mortality ratio as fishing mortality normalized by natural mortality (*F/M*). When comparing multiple stocks with different natural mortalities, the mortality ratio can better reflect the impact of fishing pressure for cross-stock analyses.

To examine whether life history traits, long-term mean and variability of fishing and temperature have influenced the relative contribution of fishing and temperature effect, we first used simple univariate regression analysis to test each variable. We then build a linear mixed effect model (LMM) with the same variable as fixed effect and habitat as random effect to check if the observed pattern remains.

### Comparing the performance of univariate SBIs to variation partitioning

To demonstrate the efficiency of variation partitioning, we compared the *P* value of explained fraction in the variation partitioning to the *P* value in regression with univariate SBIs. The univariate SBIs considered in the analysis were: 95% percentile of length class (L_95_), mean length, Shannon diversity, Pielou’s evenness index, and skewness. After calculating the univariate SBIs, each SBI was fitted to a regression model where *SBI ~ temperature* + *fishing* + temperature*fishing. If the *P* value of variation partitioning was lower than that of multilinear regression, it would suggest that variation partitioning is more efficient than univariate SBIs in terms of rejecting the null hypothesis.

### Computation and Data availability

All computation was done with R version 3.3.3. Variation partitioning was carried out using R package vegan^55^. The linear mixed effect model was done with R package lme4^56^. Further model testing and model diagnostics were done with R package lmerTest^57^. The original data (except few stocks via personal communications, see Table S1 for details), the R scripts to carry out all analyses, and the outputs are available at: https://zenodo.org/record/1211120.

## Electronic supplementary material

Supplementary Information
