# Supplementary material for: Fishing and temperature effects on the size structure of exploited fish stocks
Source: Sci Rep. 2018 May 8;8:7132. doi: 10.1038/s41598-018-25403-x (PMC5940859; doi:10.1038/s41598-018-25403-x)
Supplement: Supplementary file 1 — Supplementary Information [file 41598_2018_25403_MOESM1_ESM.docx]

**Supplementary Information**

**Fishing and Temperature effects on the size structure of exploited fish stocks**

Chen-Yi Tu^1^, Kuan-Ting Chen^1^, Chih-hao Hsieh^1,2,3,4*^

^1^Institute of Oceanography, National Taiwan University, Taipei, Taiwan

^2^Institute of Ecology and Evolutionary Biology, Department of Life Science, National Taiwan University, Taipei, Taiwan.

^3^Research Center for Environmental Changes, Academia Sinica, Taipei, Taiwan

^4^National Center for Theoretical Sciences, Taipei, Taiwan

Correspondence:

Chih-hao Hsieh

Email: [chsieh@ntu.edu.tw](mailto:chsieh@ntu.edu.tw)

**Supplementary Table S1.** Data source for size structure, fishing mortality, and life history traits of each stock. For Alaska, the abbreviation in the bracket indicates the locations of stock used as a management unit: AI, Aleutian Island; GOA, Gulf of Alaska; BSAI, Bering Sea and Aleutian Islands

| **Species** | **Common Name** | **Length composition** | **Fishing mortality/Exploitation** | **A50** | **L50** | **Linf** | **K** | **M** |
| --- | --- | --- | --- | --- | --- | --- | --- | --- |
| *Atheresthes stomias* | Arrowtooth flounder* | From PacFIN database for fillet fishery^1^ | Exploitation rate^1^ | ^1^ | ^1^ | ^1^ | ^1^ | ^1^ |
| *Sebastes goodie* | Chilipepper rockfish | Trawl fishery^2^ | Exploitation rate^2^ | ^3^ | ^3^ | ^3^ | ^3^ | ^2^ |
| *Sebastes crameri* | Dark blotched rockfish | Fisheries observation^4^ | Exploitation rate^4^ | ^5^ | ^5^ | ^6^ | ^6^ | ^4^ |
| *Microstomus pacificus* | Dover Sole | Fishery in northern and southern region combined^7^ | Exploitation rate^7^ | ^8^ | ^8^ | ^8^ | ^8^ | ^7^ |
| *Parophrys vetulus* | English Sole | Fishery from the north (N=42) region^9^ | Exploitation rate^9^ | ^10^ | ^10^ | ^11^ | ^9^ | ^9^ |
| *Ophiodon elongatus* | Lingcod | Fishery observation at north^12^ | Exploitation rate^12^ | ^13^ | ^13^ | ^11^ | ^11^ | ^12^ |
| *Sebastolobus altivelis* | Longspine thornyhead | Coast-wide length composition from the commercial landings in California (CA)^14^ | Exploitation rate^14^ | ^14^ | ^14^ | ^14^ | ^14^ | ^14^ |
| *Eopsetta jordani* | Petrale sole | Fishery length observation^15^ | Exploitation rate^15^ | ^16^ | ^16^ | ^15^ | ^15^ | ^15^ |
| *Sardinops sagax* | Sardine* | Fishery length observations at north^17^ | Exploitation rate^17^ | ^17^ | ^17^ | ^17^ | ^17^ | ^17^ |
| *Sebastes diploproa* | Splitnose rockfish | California (CA) domestic trawl fishery observation^18^ | Exploitation rate^18^ | ^19^ | ^19^ | ^5^ | ^5^ | ^18^ |
| *Sebastes ruberrimus* | Yelloweye rockfish | Length composition at port and observer^20^ | Exploitation rate^20^ | ^21^ | ^21^ | ^20^ | ^20^ | ^20^ |
| *Gadus chalcogramma* | Walleye pollock (AI)* | AI triennial survey^22^ | Exploitation rate^23^ | ^23^ | ^24^ | ^23^ | ^23^ | ^23^ |
| *Gadus chalcogramma* | Walleye pollock (GOA) | Shelikof Strait surveys in 1981-2010^25^ | Exploitation rate^26^ | ^26^ | ^26^ | ^27^ | ^27^ | ^26^ |
| *Hippoglossoides elassodon* | Flathead sole (EBS)* | EBS survey size composition^28^ | Fishing mortality estimates from selected (base) model^28^ | ^28^ | ^28^ | ^28^ | ^28^ | ^28^ |
| *Hippoglossoides elassodon* | Flathead sole (GOA)* | GOA trienniel survey size composition^29^ | Fishing mortality from the preferred (Alternative 1) model^29^ | ^30^ | ^30^ | ^30^ | ^30^ | ^29^ |
| *Gadus macrocephalus* | Pacific cod (EBS) | Length frequencies for post 1981 EBS trawl survey^31^ | Estimates of fishing mortality rates by Model B1^31^ | ^24^ | ^24^ | ^32^ | ^32^ | ^31^ |
| *Gadus macrocephalus* | Pacific cod (GOA) | Length frequencies for sub27 (cm) + 2plus (cm) trawl survey by length bin 1984-2009^33^ | Estimates of fishing mortality rates by Model B1^33^ | ^34^ | ^34^ | ^34^ | ^34^ | ^33^ |
| *Glyptocephalus zachirus* | Rex sole (GOA) | GOA trienniel survey size composition^35^ | Fishing pressure from final model estimates^35^ | ^36^ | ^36^ | ^36^ | ^36^ | ^35^ |
| *Gadus morhua* | Cod | NS-IBTS^37^ | Fishing pressure of age 2-4^38^ | ^39^ | ^39^ | ^39^ | ^39^ | ^40^ |
| *Melanogrammus aeglefinus* | Haddock | NS-IBTS^37^ | Fishing pressure of age 2-4^41^ | ^39^ | ^39^ | ^39^ | ^39^ | ^40^ |
| *Clupea harengus* | Herring | NS-IBTS^37^ | Fishing pressure of age 2-6^42^ | ^43^ | ^43^ | ^44^ | ^44^ | ^45^ |
| *Scomber scombrus* | Mackerel | NS-IBTS^37^ | Fishing pressure of age 4-8^46^ | ^47^ | ^47^ | ^39^ | ^39^ | ^48^ |
| *Trisopterus esmarkii* | Norway pout | NS-IBTS^37^ | Fishing pressure of age 1-2^49^ | ^39^ | ^39^ | ^39^ | ^39^ | ^50^ |
| *Pleuronectes platessa* | Plaice | NS-IBTS^37^ | Fishing mortality of age 2-6^51^ | ^39^ | ^39^ | ^39^ | ^39^ | ^40^ |
| *Pollachius virens* | Saithe | NS-IBTS^37^ | Fishing pressure of age 4-7^52^ | ^39^ | ^39^ | ^39^ | ^39^ | ^40^ |
| *Solea solea* | Sole | NS-IBTS^37^ | Fishing mortality of age 2-6^53^ | ^54^ | ^54^ | ^54^ | ^54^ | ^40^ |
| *Sprattus sprattus* | Sprat | NS-IBTS^37^ | Fishing pressure of age 1-2^55^ | ^11^ | ^11^ | ^56^ | ^56^ | ^45^ |
| *Merlangius merlangius* | Whiting | NS-IBTS^37^ | Fishing pressure of age 2-6^57^ | ^39^ | ^39^ | ^39^ | ^39^ | ^58^ |

*Female and male has different age at maturation (A_50_), length at maturation (L_50_), L_inf_ and K. We only use female in the analysis.

**Supplementary Table S2** Life history traits, natural mortality (M), mean of mortality ratio (meanF_M), temperature (mean T) and CV (CV of F_M, CV of T) for the analyzed species/stocks.

| **Species** | **Habitat** | **Linf** | **K** | **A50** | **L50** | **M** | **Mean F_M** | **Mean T** | **CV of F_M** | **CV of T** |
| --- | --- | --- | --- | --- | --- | --- | --- | --- | --- | --- |
| *Atheresthes stomias* | demersal | 72.26 | 0.17 | 4.00 | 37.30 | 0.166 | 0.618 | 13.079 | 0.321 | 0.033 |
| *Sebastes goodie* | demersal | 56.60 | 0.20 | 3.00 | 26.00 | 0.16 | 0.664 | 13.046 | 0.588 | 0.031 |
| *Sebastes crameri* | demersal | 39.18 | 0.09 | 4.00 | 27.00 | 0.07 | 1.171 | 13.008 | 0.662 | 0.033 |
| *Microstomus pacificus* | demersal | 47.40 | 0.09 | 7.00 | 31.10 | 0.09 | 1.198 | 12.927 | 0.402 | 0.034 |
| *Parophrys vetulus* | demersal | 57.00 | 0.30 | 4.00 | 23.00 | 0.26 | 0.542 | 12.937 | 0.461 | 0.032 |
| *Ophiodon elongatus* | demersal | 104.00 | 0.20 | 3.80 | 55.70 | 0.18 | 0.444 | 12.997 | 0.755 | 0.032 |
| *Sebastolobus altivelis* | bathydemersal | 31.20 | 0.06 | 11.00 | 17.80 | 0.06 | 0.596 | 13.040 | 0.778 | 0.034 |
| *Eopsetta jordani* | demersal | 56.24 | 0.14 | 5.15 | 33.10 | 0.2 | 1.923 | 12.892 | 0.199 | 0.031 |
| *Sardinops sagax* | pelagic | 30.00 | 0.91 | 1.25 | 16.00 | 0.4 | 0.413 | 13.014 | 0.603 | 0.035 |
| *Sebastes diploproa* | bathydemersal | 34.18 | 0.13 | 7.00 | 19.00 | 0.048 | 0.401 | 13.009 | 0.787 | 0.033 |
| *Sebastes ruberrimus* | demersal | 62.38 | 0.49 | 11.60 | 38.78 | 0.047 | 1.457 | 13.036 | 0.623 | 0.031 |
| *Gadus chalcogramma* (AI) | benthopelagic | 58.69 | 0.34 | 4.50 | 39.00 | 0.2 | 0.634 | 4.308 | 1.120 | 0.058 |
| *Gadus chalcogramma* (GOA) | benthopelagic | 57.70 | 0.32 | 4.90 | 43.00 | 0.3 | 0.358 | 5.537 | 0.382 | 0.077 |
| *Hippoglossoides elassodon* (EBS) | demersal | 44.60 | 0.10 | 8.70 | 24.00 | 0.2 | 0.222 | 2.367 | 0.305 | 0.282 |
| *Hippoglossoides elassodon* (GOA) | demersal | 48.86 | 0.16 | 8.74 | 33.30 | 0.2 | 0.069 | 5.486 | 0.469 | 0.095 |
| *Gadus macrocephalus* (EBS) | demersal | 104.30 | 0.22 | 5.80 | 67.00 | 0.34 | 0.134 | 2.347 | 0.409 | 0.314 |
| *Gadus macrocephalus* (GOA) | demersal | 156.72 | 0.08 | 4.30 | 50.00 | 0.38 | 0.206 | 5.660 | 0.738 | 0.102 |
| *Glyptocephalus zachirus* (GOA) | demersal | 41.82 | 0.39 | 5.10 | 35.20 | 0.17 | 0.539 | 5.432 | 0.504 | 0.071 |
| *Gadus morhua* | demersal | 123.10 | 0.23 | 3.80 | 69.70 | * | 2.179 | 6.118 | 0.276 | 0.893 |
| *Melanogrammus aeglefinus* | demersal | 68.30 | 0.19 | 2.50 | 33.50 | * | 0.873 | 6.118 | 0.447 | 1.210 |
| *Clupea harengus* | benthopelagic | 33.60 | 0.33 | 3.00 | 25.30 | * | 0.952 | 6.118 | 0.448 | 2.732 |
| *Scomber scombrus* | pelagic | 39.90 | 0.36 | 1.50 | 25.00 | 0.15 | 1.906 | 6.185 | 0.249 | 2.652 |
| *Trisopterus esmarkii* | benthopelagic | 23.00 | 0.52 | 2.30 | 19.00 | * | 1.158 | 6.263 | 0.784 | 1.671 |
| *Pleuronectes platessa* | demersal | 54.50 | 0.11 | 2.50 | 26.60 | 0.1 | 5.105 | 6.118 | 0.334 | 1.506 |
| *Pollachius virens* | demersal | 177.10 | 0.07 | 4.60 | 55.40 | 0.2 | 2.533 | 6.118 | 0.250 | 1.517 |
| *Solea solea* | demersal | 45.30 | 0.36 | 4.00 | 28.90 | 0.1 | 5.261 | 6.118 | 0.194 | 1.461 |
| *Sprattus sprattus* | pelagic | 15.00 | 0.50 | 2.00 | 11.50 | * | 3.007 | 6.118 | 0.502 | 0.714 |
| *Merlangius merlangius* | demersal | 42.40 | 0.32 | 1.50 | 20.20 | * | 0.597 | 6.416 | 0.522 | 1.703 |

* The estimates of natural mortality were under continuous revising for management purpose during the data period for these stocks. Please refer to original reference (see Table S1) or dataset compiled by us (https://zenodo.org/record/1210792) for details.


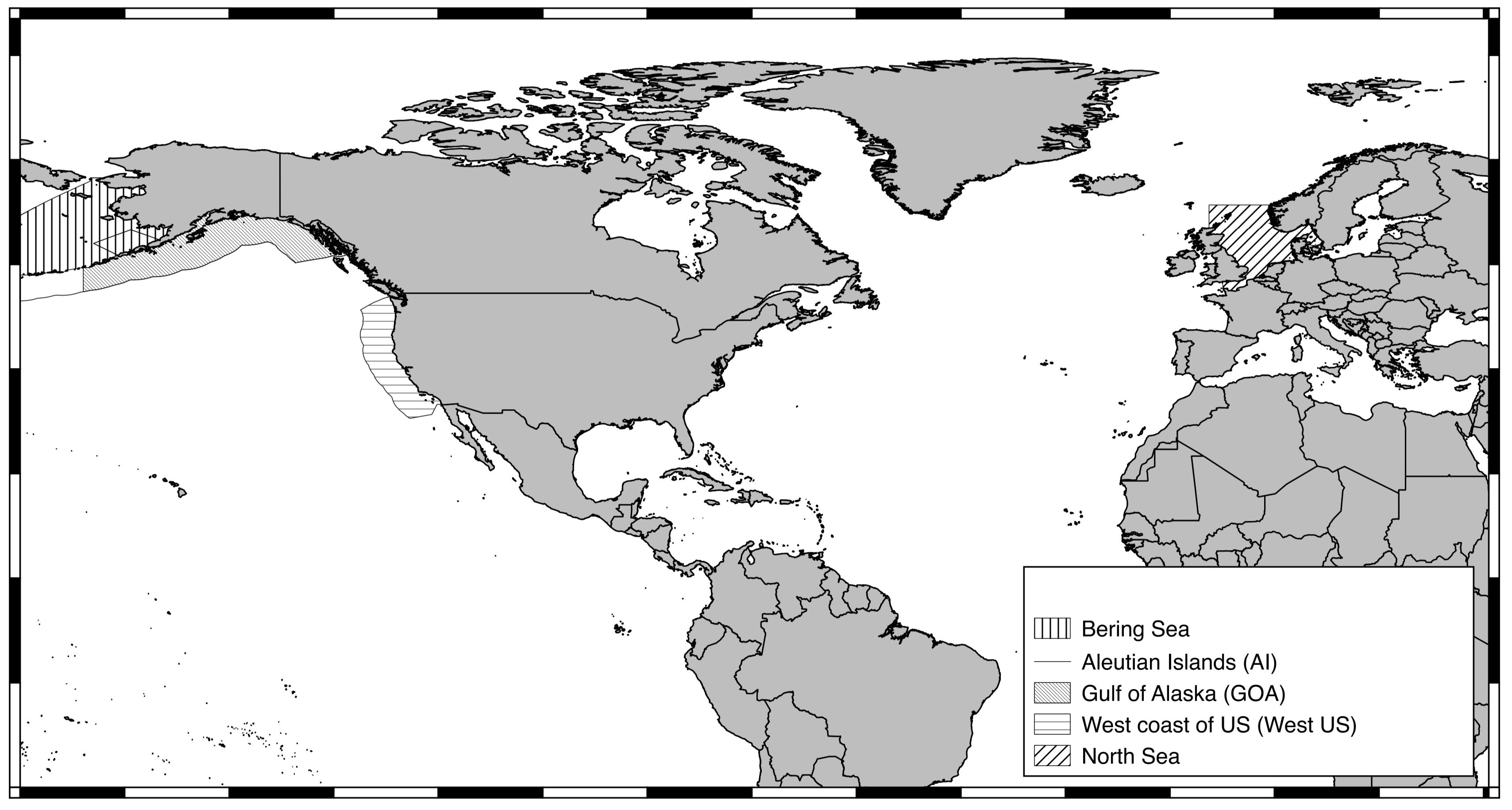


**Supplementary Figure S1.** Fishing areas defined in the analysis. This map is generated with QGIS 2.18.13 “Las Palmas” on Mac OS X (Available online at http://www.kyngchaos.com/software/qgis)

**Supplementary Figure S2.** Time series of temperature used in the analysis. For the west coast of US, we used the average sea surface temperature. For other regions, we used the average of bottom temperature from the trawl survey. The dots in AI and GOA represent the sampling years.

**Supplementary Figure S3.** Time series of surface temperature measurements in AI, EBS, GOA and North Sea. Surface temperature of AI, EBS and GOA were measured during bottom trawl survey. The North Sea surface temperature is all data collected at depths of <10m from reduced CTD, bottle, underway/pump and mooring data in Marsden Square no. 216 (North Sea) via <http://ocean.ices.dk/data/surface/surface.htm>. The dots in AI and GOA represent the sampling years.

**Supplementary Figure S4.** Explained variation by fishing in relation to the life history traits. The dash line is the best- fitted regression line based on LMM.

**Supplementary Figure S5.** Explained variation by fishing in relation to the mean and CV of mortality ratio or CV of temperature. The dash line is the best-fitted regression line based on LMM.

**Supplementary Figure S6.** Explained variation by temperature in relation to life history traits. The dash line is the best-fitted regression line based on LMM.

**Supplementary Figure S7.** Explained variation by fishing in relation to the mean and CV of mortality ratio or CV of temperature. The dash line is the best-fitted regression line based on LMM.

**References**

1 Kaplan, I. C. & Helser, T. E. Stock assessment of the arrowtooth flounder (*Atheresthes stomias*) population off the west coast of the United States in 2007. (Pacific Fishery Management Council, 2007).

2 Field, J. C. Status of the Chilipepper rockfish, *Sebastes goodei*, in 2007. (2007).

3 Wilkins, M. E. Size composition, age composition, and growth of chilipepper, *Sebastes goodei*, and bocaccio, *S. paucispinis*, from the 1977 rockfish survey. *Mar. Fish. Rev.* **42**, 48-53 (1980).

4 Wallace, J. R. & Hamel, O. S. Status and future prospects for the darkblotched rockfish resource in waters off Washington, Oregon, and California as updated in 2009. 97220-91384 (2009).

5 Echeverria, T. W. Thirty-four species of California rockfishes: maturity and seasonality of reproduction. *Fishery Bulletin* **85** (1987).

6 Archibald, C., Leaman, B., Station, P. B. & Shaw, W. *Growth and mortality estimates of rockfishes (Scorpaenidae) from BC coastal waters, 1977-1979*. (Nanaimo, BC: Government of Canada, Fisheries and Oceans, 1981).

7 Sampson, D. B. The status of Dover sole off the US West Coast in 2005. *Pacific Fisheries Management Council, Portland, OR* (2005).

8 Hunter, J. R., Butler, J. L., Kimbrell, C. & Lynn, E. A. Bathymetric patterns in size, age, sexual maturity, water content, and caloric density of Dover sole, Microstomus pacificus. *CalCOFI Reports* **31**, 132-144 (1990).

9 Stewart, I. J. Updated US English sole stock assessment: Status of the resource in 2007. (2007).

10 Sampson, D. B. & Al‐Jufaily, S. Geographic variation in the maturity and growth schedules of English sole along the US west coast. *Journal of Fish Biology* **54**, 1-17 (1999).

11 Froese, R. & Pauly, D. *FishBase*, <<http://www.fishbase.org/>> (2016).

12 Hamel, O. S., Sethi, S. A. & Wadsworth, T. F. Status and future prospects for lingcod in waters off Washington, Oregon, and California as assessed in 2009. 565-626 (2009).

13 Silberberg, K. R., Laidig, T. E., Adams, P. B. & Albin, D. Analysis of maturity in lingcod, Ophiodon elongatus. *California Fish and Game* **87**, 139-152 (2001).

14 Fay, G. Stock assessment and status of longspine thornyhead (Sebastolobus altivelis) off California, Oregon and Washington in 2005. *Pacific Fisheries Management Council, Portland, OR* (2005).

15 Haltuch, M. A. & Hicks, A. Status of the US petrale sole resource in 2008. (2009).

16 Hannah, R. W., Parker, S. J. & Fruh, E. L. Length and age at maturity of female petrale sole (*Eopsetta jordani*) determined from samples collected prior to spawning aggregation. *Fishery Bulletin* **100**, 711-719 (2002).

17 Hill, K. T. *et al.* Assessment of the Pacific sardine resource in 2007 for US management in 2008. (2007).

18 Gertseva, V. V., Cope, J. M. & Pearson, D. E. Status of the US splitnose rockfish (*Sebastes diploproa*) resource in 2009. (Northwest Fisheries Science Center, NOAA Fisheries, 2009).

19 Boehlert, G. & Kappenman, R. Variation of growth with latitude in two species of rockfish (*Sebastes pinniger* and *S. diploproa*) from the northeast Pacific Ocean. *Mar. Ecol. Prog. Ser* **3**, 1-10 (1980).

20 Stewart, I. J., Wallace, J. R. & McGilliard, C. Status of the US yelloweye rockfish resource in 2009. (2009).

21 Hannah, R. W., Blume, M. T. & Thompson, J. E. *Length and age at maturity of female yelloweye rockfish (Sebastes rubberimus) and cabezon (Scorpaenichthys marmoratus) from Oregon waters based on histological evaluation of maturity*. (Oregon Department of Fish and Wildlife, Marine Resources Program, 2009).

22 Personal communication from Steve Barbeaux, research fishery scientist at Alaska Fisheries Science Center. Email: steve.barbeaux@noaa.gov

23 Barbeaux, S., Ianelli, J., Gaichas, S. & Wilkins, M. A: Assessment of the Pollock stock in the Aleutian Islands. (2009).

24 Witherell, D. Groundfish of the Bering Sea and Aleutian Islands area: species profiles 2001. *North Pacific Fishery Management Council. Anchorage, AK* **99501** (2000).

25 Personal communication from Martin Dorn, research fishery scientist at Alaska Fisheries Science Center. Email: Martin.Dorn@noaa.gov

26 Dorn, M. *et al.* Assessment of walleye pollock in the Gulf of Alaska. *Stock assessment and fishery evaluation report for the groundfish resources of the Gulf of Alaska*, 53-156 (2010).

27 Megrey, B. A. Population dynamics and management of walleye pollock (Theragra chalcogramma) in the Gulf of Alaska, 1976–1986. *Fisheries Research* **11**, 321-354, doi:<http://dx.doi.org/10.1016/0165-7836(91)90008-4> (1991).

28 Stockhausen, W. T., Nichol, D. & Palsson, W. Assessment of the flathead sole stock in the Bering Sea and Aleutian Islands. (2012).

29 Stockhausen, W. T., Wilkins, M. E. & Martin, M. H. 8. Assessment of the Flathead Sole Stock in the Gulf of Alaska. 681-742 (North Pacific Fishery Management Council, Anchorage, AK, 2009).

30 Stark, J. W. A comparison of the maturation and growth of female flathead sole in the central Gulf of Alaska and south‐eastern Bering Sea. *Journal of fish biology* **64**, 876-889 (2004).

31 Thompson, G. G., Ianelli, J. & Lauth, R. R. Chapter 2: Assessment of the Pacific cod stock in the eastern Bering Sea and Aleutian Islands area. 235-440 (2009).

32 Kimura, D., Shimada, A. & Lowe, S. Estimating von Bertalanffy growth parameters of sablefish Anoplopoma fimbria and Pacific cod Gadus macrocephalus using tag-recapture data. *Fishery Bulletin* **91**, 271-280 (1993).

33 Thompson, G. G., Ianelli, J. & Wilkins, M. Assessment of the Pacific cod stock in the Gulf of Alaska. 165-352 (2009).

34 Stark, J. W. Geographic and seasonal variations in maturation and growth of female Pacific cod (Gadus macrocephalus) in the Gulf of Alaska and Bering Sea. *Fishery Bulletin* **105**, 396-407 (2007).

35 Stockhausen, W. T., Wilkins, M. E. & Martin, M. H. 6. Assessment of the Rex Sole Stock in the Gulf of Alaska. 629-690 (North Pacific Fishery Management Council, Anchorage, AK, 2009).

36 Abookire, A. A. Reproductive biology, spawning season, and growth of female rex sole (Glyptocephalus zachirus) in the Gulf of Alaska. *Fishery Bulletin* **104**, 350-359 (2006).

37 ICES. *North Sea International Bottom Trawl Survey (1977-2014)*, <<http://datras.ices.dk>> (

38 ICES. *Cod (Gadus morhua) in Subarea IV and Divisions VIId and IIIa West (North Sea, Eastern English Channel, Skagerrak)* < <http://standardgraphs.ices.dk/ViewCharts.aspx?key=8052>> (2016).

39 Jennings, S., Reynolds, J. D. & Mills, S. C. Life history correlates of responses to fisheries exploitation. *Proceedings of the Royal Society of London. Series B: Biological Sciences* **265**, 333-339, doi:10.1098/rspb.1998.0300 (1998).

40 ICES. Report of the Working Group on the Assessment of Demersal Stocks in the North Sea and Skagerrak (WGNSSK). 19pp (Hamburg, Germany, 2017).

41 ICES. *Haddock in Subarea IV and Divisions IIIa West and VIa (North Sea, Skagerrak and West of Scotland)* <<http://standardgraphs.ices.dk/ViewCharts.aspx?key=8068>> (2016).

42 ICES. *Herring in Subarea IV and Divisions IIIa and VIId (North Sea autumn spawners)* <<http://standardgraphs.ices.dk/ViewCharts.aspx?key=7689>> (2016).

43 ICES. ICES FishMap Species fact sheets- Herring (Clupea harengus). (2006).

44 Kienzle, M. Estimation of the Parameters of von Bertalanffy Growth Function for teh Main Commerical Species of the North Sea estimation of the parameters of von Bertalanffy growth function for the main commercial species of the North Sea. (2005).

45 ICES. Report of the Herring Assessment Working Group for the Area South of 62 ̊N (HAWG). 867 (ICES HQ, Copenhagen, Denmark, 2016).

46 ICES. *Mackerel in the Northeast Atlantic (combined Southern, Western and North Sea spawning components)* <<http://standardgraphs.ices.dk/ViewCharts.aspx?key=8120>> (2016).

47 ICES. ICES FishMap Species fact sheets- Mackerel (Scomber scombrus). (2006).

48 ICES. Report of the Working Group on Widely Distributed Stocks (WGWIDE). 500 (ICES HQ, Copenhagen, Denmark, 2016).

49 ICES. *Norway Pout in Subarea IV (North Sea) and IIIa (Skagerrak - Kattegat) - Autumn assessment* <<http://standardgraphs.ices.dk/ViewCharts.aspx?key=7998> > (2016).

50 ICES. Report of the Benchmark Workshop on Norway Pout (Trisopterus esmarkii) in Subarea 4 and Division 3a (North Sea, Skagerrak, and Kattegat). 69 (Copenhagen, Denmark, 2017).

51 ICES. *Plaice Subarea IV (North Sea)* <<http://standardgraphs.ices.dk/ViewCharts.aspx?key=7445>> (2016).

52 ICES. *Saithe in Subarea IV (North Sea) Division IIIa West (Skagerrak) and Subarea VI (West of Scotland and Rockall)*, <<http://standardgraphs.ices.dk/ViewCharts.aspx?key=8066>> (2016).

53 ICES. *Sole in Subarea IV (North Sea)*, <<http://standardgraphs.ices.dk/ViewCharts.aspx?key=7722>> (2016).

54 de Veen, J. F. On changes in some biological parameters in the North Sea sole (Solea solea L.). *ICES Journal of Marine Science* **37**, 60-90, doi:10.1093/icesjms/37.1.60 (1976).

55 ICES. *Sprat in Subarea IV (North Sea)* <<http://standardgraphs.ices.dk/ViewCharts.aspx?key=7181>> (2016).

56 Froese, R. & Sampang, A. Potential indicators and reference points for good environmental status of commercially exploited marine fishes and invertebrates in the German EEZ. (2013).

57 ICES. *Whiting Subarea IV (North Sea) and Division VIId (Eastern Channel)* <<http://standardgraphs.ices.dk/ViewCharts.aspx?key=7483>> (2016).

58 ICES. Report of the Inter-Benchmark Protocol for Whiting in the North Sea (IBP Whiting). 119 (By correspondence, 2016).
